# Robot-Assisted Atrial Septal Defect Closure Via the Left Atrium: Dual Case Reports

**DOI:** 10.1177/15569845241296083

**Published:** 2024-11-12

**Authors:** Yazan N. AlJamal, Hiroto Kitahara, Husam H. Balkhy

**Affiliations:** 1 Section of Cardiac Surgery, Department of Surgery, University of Chicago, IL, USA

## Case Presentation

We present 2 patients with secundum atrial septal defect (ASD) who underwent totally endoscopic robot-assisted ASD closure via the left atrium (Supplemental Video). Our operating room setup, port size, location, and peripheral cardiopulmonary bypass configuration were published previously.^1^

Once cardiopulmonary bypass was initiated, the pericardium was opened, and a temporary pacing wire was placed on the right ventricle. The patient was cooled to 31 °C. Rapid ventricular pacing (800 bpm) was performed to achieve a stable fibrillary arrest. Snares were placed around the cavae.

A left atriotomy was made along Sondergaard’s groove, and the robotic dynamic atrial retractor was positioned to expose the ASD. Two percutaneous drains were placed into the left ventricle and the left pulmonary veins, respectively. In patient 1, there was a large secundum defect and multiple fenestrations. The thin secundum tissue was sharply resected, and a PhotoFix ultrathin bovine pericardial patch was sewn onto the edges of the fossa ovalis with a running 4-0 Prolene suture. In patient 2, the defect extended fairly low toward the inferior vena cava (IVC). A PhotoFix bovine pericardial patch was sewn onto the edges of the defect with a running 4-0 Prolene suture. Right atrial filling confirmed no evidence of residual defects in the septum. The left atriotomy was closed with a 4-0 Prolene suture while the patient was rewarmed and deairing was completed. The left ventricular drain was maintained to facilitate further deairing after spontaneous conversion to sinus rhythm. Postoperative transesophageal echocardiogram showed no evidence of atrial-level shunt by either agitated bubble study or a Doppler study. Both patients were discharged on postoperative day 1 and were doing well with complete resolution of symptoms at the 30-day follow-up visit.

## Discussion

ASD closure through median sternotomy or right thoracotomy can be associated with disadvantages including postoperative pain and wound complications. Percutaneous closure is appealing and has been widely adopted because it is significantly less invasive. However, it is not suitable for all patients (e.g., those with very large ASDs or insufficient surrounding rim, multiple defects, an atrial septal aneurysm, and in patients with nickel allergy). In addition, long-term complications of septal closure devices with erosion into vital structures has been reported.^2^

Robotic cardiac surgery has been shown to be safe, effective,^3,4^ and by far the least invasive approach for surgical ASD closure, especially when performed endoscopically.^5^ By avoiding large incisions and rib spreading, it offers less perioperative morbidity and faster recovery.^6^

The standard approach for ASD closure is via a right atriotomy. Closing an ASD through a left atriotomy has certain advantages and has never been reported in the literature when done using a robotic approach. Within our multispectrum robotic endoscopic program,^7^ we discovered the excellent exposure to a concomitant ASD from the left atrium (LA) during mitral valve (MV) repair and have started using this approach in patients with isolated ASD. There are some advantages to using an LA approach for ASD closure that can potentially simplify the procedure (Supplemental Table):

1. The defect and its surrounding structures (including coronary sinus, MV, IVC rims) can be visualized more comprehensively from the LA using a 30° (up) scope, which has the potential of lowering the risk of coronary sinus and IVC injury.
2. A single long femoral venous cannula can be used, thus simplifying the operation.
3. Deairing is more straightforward using an LA approach.
4. In selected patients, ventricular fibrillatory arrest can simplify the operation by avoiding aortic manipulation and is our preferred method in robotic totally endoscopic intracardiac surgery in patients with small femoral arteries. It avoids using larger arterial cannulae necessary for endoaortic balloon occlusion. It has been found to be safe and effective in minimally invasive MV surgery.^8^ The most important consideration when using this technique is adequate deairing to prevent systemic embolization, as detailed in our recent publication.^7^ In addition to flooding the field with continuous low carbon dioxide insufflation and maintaining high systemic blood pressure (to avoid air from entering the aortic root), the left ventricle is kept empty and the MV incompetent throughout the procedure by maintaining a vent across the valve. We believe that meticulous air-removal maneuvers are made more feasible via a left atriotomy.

In conclusion, ASD closure via a left atriotomy using a robotic totally endoscopic approach is safe and feasible and has certain advantages. Patient selection and attention to detail during deairing are important to the success of the procedure when using ventricular fibrillation arrest as demonstrated in these 2 cases. The choice of approach depends on surgeon preference and experience and individual patient-specific considerations.

## Supplemental Material

sj-pdf-1-inv-10.1177_15569845241296083 – Supplemental material for Robot-Assisted Atrial Septal Defect Closure Via the Left Atrium: Dual Case Reports Supplemental material, sj-pdf-1-inv-10.1177_15569845241296083 for Robot-Assisted Atrial Septal Defect Closure Via the Left Atrium: Dual Case Reports by Yazan N. AlJamal, Hiroto Kitahara and Husam H. Balkhy in Innovations
