# Supplementary material for: Robot-Assisted Atrial Septal Defect Closure Via the Left Atrium: Dual Case Reports
Source: Innovations (Phila). 2024 Nov 12;19(6):672–3. doi: 10.1177/15569845241296083 (PMC11663434; doi:10.1177/15569845241296083)
Supplement: sj-pdf-1-inv-10.1177_15569845241296083 – Supplemental material for Robot-Assisted Atrial Septal Defect Closure Via the Left Atrium: Dual Case Reports [file sj-pdf-1-inv-10.1177_15569845241296083.pdf]

Supplemental Table: Utility of Robot-Assisted Atrial Septum Defect Closure Via Left Atrium Versus Right Atrium Approaches.

	**Left atrium approach**	**Right atrium approach**
Visualization of the defect and its surrounding structure (including coronary sinus, mitral valve, inferior vena cava rims)	+++	+
Utilizing single venous cannulation	+	N/A (require 2 venous cannulas)
Deairing the left ventricle	+++	N/A
